# Exploring the role of non-pharmaceutical interventions (NPIs) in flattening the Greek COVID-19 epidemic curve

**DOI:** 10.1038/s41598-021-90293-5

**Published:** 2021-06-03

**Authors:** Amaryllis Mavragani, Konstantinos Gkillas

**Affiliations:** 1 Department of Computing Science and Mathematics, Faculty of Natural Sciences, University of Stirling, Stirling, Scotland FK9 4LA UK; 2 Department of Management Science and Technology, University of Patras, Patras, Greece

**Keywords:** Health policy, Public health, Epidemiology, Computational models, Statistical methods

## Abstract

Due to the COVID-19 pandemic originating in China in December 2019, apart from the grave concerns on the exponentially increasing casualties, the affected countries are called to deal with severe repercussions in all aspects of everyday life, from economic recession to national and international movement restrictions. Several regions managed to handle the pandemic more successfully than others in terms of life loss, while ongoing heated debates as to the right course of action for battling COVID-19 have divided the academic community as well as public opinion. To this direction, in this paper, an autoregressive COVID-19 prediction model with heterogeneous explanatory variables for Greece is proposed, taking past COVID-19 data, non-pharmaceutical interventions (NPIs), and Google query data as independent variables, from the day of the first confirmed case—February 26th—to the day before the announcement for the quarantine measures’ softening—April 24th. The analysis indicates that the early measures taken by the Greek officials positively affected the flattening of the epidemic curve, with Greece having recorded significantly decreased COVID-19 casualties per million population and managing to stay on the low side of the deaths over cases spectrum. In specific, the prediction model identifies the 7-day lag that is needed in order for the measures’ results to actually show, i.e., the optimal time-intervention framework for managing the disease’s spread, while our analysis also indicates an appropriate point during the disease spread where restrictive measures should be applied. Present results have significant implications for effective policy making and in the designing of the NPIs, as the second wave of COVID-19 is expected in fall 2020, and such multidisciplinary analyses are crucial in order to understand the evolution of the Daily Deaths to Daily Cases ratio along with its determinants as soon as possible, for the assessment of the respective domestic health authorities’ policy interventions as well as for the timely health resources allocation.

## Introduction

In December 2019, a novel coronavirus that causes severe acute respiratory syndrome (SARS) was detected in Wuhan, China^1,2^. The coronavirus is of unknown origin, though there are suggestions pointing to a wet market in said region as per the virus’ emergence^3^. With WHO declaring the new coronavirus disease—officially COVID-19 as of February 11th^4^—a pandemic on March 11th, 2020^5^, and after most countries having been affected, the importance of taking immediate action becomes evident.

To this direction, flattening the epidemic curve and exploring novel approaches in dealing with COVID-19 spreading is what has been the first priority over the past months. Italy, being the first negative example of the impact of said disease in Europe, alarmed the rest of the countries to adopt certain non-pharmaceutical interventions (NPIs), including a chain of preventive and movement restrictive measures in order to avoid the spreading of the virus. However, certain regions decided on alternative routes (e.g., Sweden^6^), possibly aiming at herd immunity and in minimizing the COVID-19’s social and economic impact.

Heated ongoing discussions and debates are dividing the scientific community and the public, as to which approach should be followed in order to combat COVID-19^7,8^. Following the case of Italy that was heavily affected—with daily deaths surpassing 300 from mid-March to the end of April and with a record of 919 on March 27th ^9^—, it is evident that COVID-19 is not that easy to handle as seemed at first.

**Figure 1:**
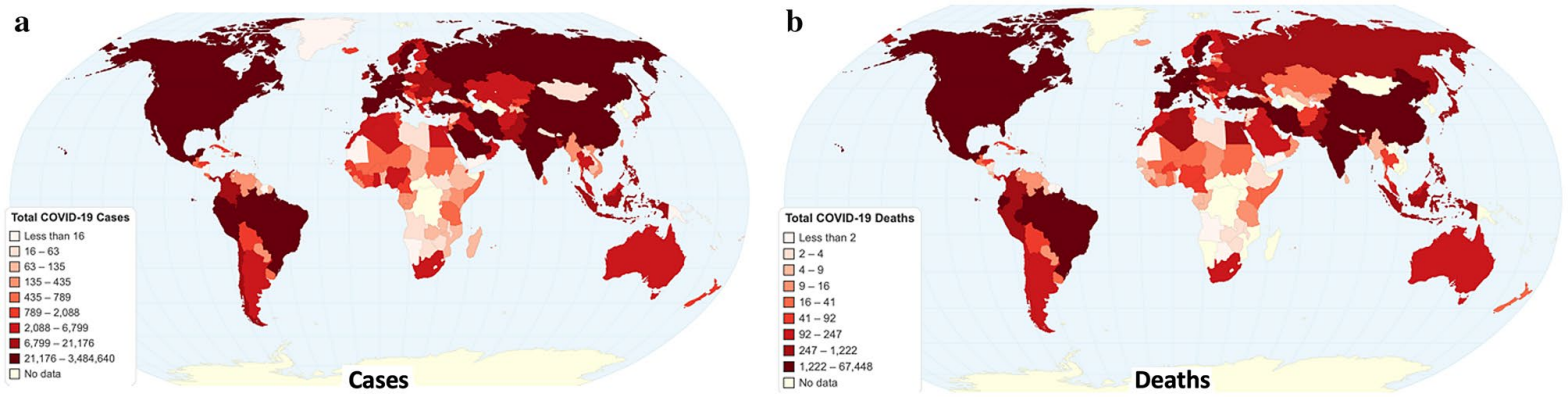
Cumulative worldwide COVID-19 (**a**) cases and (**b**) deaths (Chartsbin^22^).

**Figure 2:**
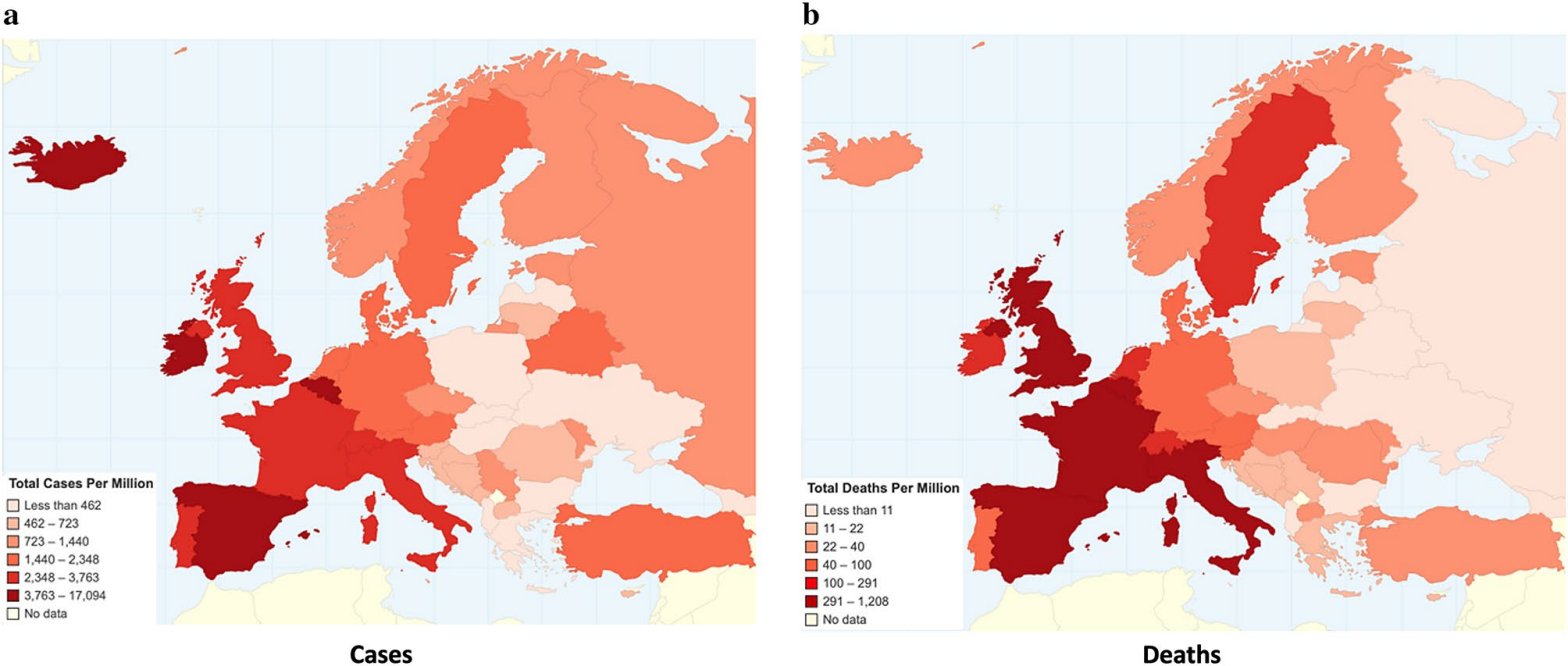
Total COVID-19 (**a**) cases and (**b**) deaths per million population in Europe (Chartsbin^22^).

Several approaches are identified, mainly those of mass population testing, like South Korea^10^ and Germany^11^, early closing of borders and movement restrictions like in Slovakia^12,13^, and few light measures, like Sweden^6^. Figure 1 depicts the cumulative cases and deaths, and Fig. 2 depicts the total cases and total deaths per million population in Europe up to May 3rd, 2020.

As countries have different populations and different approaches to testing in order to identify cases, the best available measure for comparison is deaths per million population. This redaction is done only for the purposes of the comparison in this case, as several other factors can affect the number of deaths, as, for example, population density, weather, population age, geographical location, open borders, flights per day, or socioeconomic diversity.

A very important question that should be addressed in order for the world to be ready for the second COVID-19 wave next fall^14,15^, is that of identifying the appropriate course of action in battling COVID-19. To this end, we investigate one of the most internationally discussed EU cases, that of Greece^16–19^; a country that managed to successfully deal with the first COVID-19 wave, as shown in Fig. 2.

To that end, the first question that needs to be addressed is whether or not NPI adoption can flatten the epidemic curve. If yes, the second question—and as we expect that the measures will show their effect in the decrease of cases and deaths in an unknown number of days after adoption since there is a time delay from infection to symptoms onset, to hospital admission, to recovery (or death)—, is the time frame that should be considered in order to determine if the adopted measures have started exhibiting results, i.e., the optimal time intervention framework for the NPIs.

In this paper, we suggest an autoregressive model with exogenous/explanatory variables over different interval sizes for assessing the effectives of the NPIs in Greece during the first wave of the pandemic (March–April 2020), aiming at investigating the optimal time intervention framework for the effectiveness of the adopted NPIs. What made the Greek case successful in dealing with the pandemic in terms of casualties and in maintaining the state of its national health care system, was that the Greek officials adopted NPIs at a very early stage; schools were announced to be closing just 1 day after WHO declared COVID-19 a pandemic on March 11th, and while the country had recorded less than 100 confirmed cases and no casualty—the first death was recorded the next day.

Furthermore, we extend this approach and develop two additional prediction models in order to also explore the number of days needed for the NPIs to come into effect for the Daily Cases and Daily Deaths variables independently. From a methodology perspective, the novelty of this approach lies in its multidisciplinarity. We focus not only on past COVID-19 data on cases and deaths, but we also take into account the active cases, the recovered cases, Google query data, and, most importantly, the role of NPIs in minimizing the spread of the virus. Such multidisciplinary analyses are essential in order to elaborate on the significance of combining various disease surveillance variables and methods, and take full advantage of the available tools, real-time worldwide data, and resources that we have at our disposal, contrary to past experiences with epidemics and pandemics.

The rest of the paper is structured as follows: “Methods” section consists of the detailed description of the data collection procedure and predictability analysis for the COVID-19 prediction models, while in “Results”, “Discussion” sections, the results are presented and discussed, respectively.

## Methods

### COVID-19

Worldwide and Europe data on COVID-19 cases and deaths were retrieved from the Worldometers platform^20^. Greek cases, deaths, and active data were retrieved from the same platform, while data for the geographical distribution of cases and deaths in Greece were retrieved from COVID-19’s page on the official Greek government platform^21^. Maps were created with Chartsbin and Mapcharts^22,23^. The selected timeframe is from February 26th to April 24th, as these two dates mark important milestones: first confirmed case and end-of-quarantine announcement, respectively. All data were collected collectively and on the same day after the ending of the examined period, in order to also account for any potential corrections during this time.

### Non-pharmaceutical interventions (NPIs)

Since no official COVID-19 cure exists, and as the vaccine is not yet available and could be more than a year before it is ready for public distribution, NPIs, as, for example, house quarantine or international flights restrictions are required in order to monitor and minimize disease spreading. The timely adoption of such measures is what made the Greek case that successful, and the statistical significance of the days of adoption are explored order to elaborate on the importance of NPIs.

Greek officials immediately acted on adopting preventive measures for the spread of the disease, with the first NPI being announced along with WHO declaring COVID-19 a pandemic on March 11th, and, effective as of March 12th, ordered the closing of all schools and universities^24^. Retail shops, gyms, cafes, restaurants, etc., followed over the next days^25,26^, while less than 2 weeks later, on March 23rd, strict movement measures took place, including house quarantine and special forms and SMS, meaning that sending an SMS or filling in a certain form was required in order to exit the household for all kinds of commuting/movement^27^. Regionally, two villages in the Kozani and one in the Xanthi Prefectures were put under complete lockdown, respectively^28,29^, while the two major cities close by were also put under special quarantine measures on March 31st^30,31^. Table 1 consists of the measures that were considered for the NPIs variable of the prediction model.

**Table 1:** Adopted measures considered in developing the NPIs variable.

Description	Date
Closing of schools and universities	March 11
Complete regional lockdown in two villages in Kozani	March 12
Closing of cafes, bars, and restaurants	March 14
Closing of retail and gyms	March 18
Special forms and SMS for commuting	March 23
Flight restrictions to and from certain countries	March 24
Strict regional lockdown in two municipalities	March 31
Strict lockdowns in certain regions	April 21

The complete-lockdown measures are of importance despite being in selected municipalities and villages, as they refer to the most affected regions of the country. In these cases, outdoor exercise was not allowed, while even caregivers were not allowed to provide assistance to the elderly or people in need (when living in different households); the latter was handled centrally. In addition, citizens were not allowed to physically attend any Easter celebrations, or Mass and Divine Services in churches during Holy Week and the week after Easter.

### Google Trends

Several approaches in the monitoring and analysis of several epidemiological characteristics of the virus have been recorded up to this point using Google Trends data^32–35^. Google Trends is an open online tool that provides information on the behavior towards selected topics and keywords. Such infodemiology variables are suggested to accurately measuring the users’ online search patterns, and, in this case, assist with exploring the public’s perception and interest towards COVID-19.

In this paper and in line with the Google Trends infodemiology methodology framework^36^, normalized Google Trends data were retrieved on a sequence of Google queries from February 26th, which is the date that the first confirmed COVID-19 case in the country was recorded, up to April 24th, which is the day before the announcement of the softening of the quarantine measures. Google describes the normalization procedure as follows: “*Search results are normalized to the time and location of a query by the following process: Each data point is divided by the total searches of the geography and time range it represents to compare relative popularity. Otherwise, places with the most search volume would always be ranked highest. The resulting numbers are then scaled on a range of 0 to 100 based on a topic’s proportion to all searches on all topics. Different regions that show the same search interest for a term don't always have the same total search volumes.*”^37^.

Google Trends is not case sensitive, but it does take into account accents, special characters, and misspellings. Greek is a rather complicated language in terms of accents and spelling, and the spelling of the translation of the word ‘coronavirus’ had not been definite. To that end, and to ensure that the majority of coronavirus searches were included in the analysis, the following procedure for the selection of the examined keywords was followed.

At first, there existed four differently spelled terms to express coronavirus, i.e., “Κορωνοιός”, “Κορωναιός”, “Κορονοιός”, and “Κοροναιός”, with all terms including accents. Each term was compared to itself without the accent in Google, and all cases exhibited non-significant results for the terms with the accents. As the popularity of the terms “Κορωναιός” and Κοροναιός”, though used during the first days of the epidemic, quickly decreased and was not widely adopted by the experts, media, and public, they exhibit significantly less interest, and thus not included in further analysis. Therefore, the Greek terms “Κορωνοιος” and “Κορονοιος” were selected at this stage and, in order to also include searches conducted in English, “Coronavirus (search term)” was also added in the analysis, i.e., data for the “κορωνοιος + κορονοιος + coronavirus” sequence of search terms were retrieved for the Google query data variable.

### Predictability analysis

A predictability analysis for COVID-19 Daily Deaths and Daily Cases ratio in Greece is performed; the prediction model is based on an autoregressive model with heterogeneous explanatory variables ($AR-{\rm H}X$). This proposed model is constructed in order to incorporate and study short-term and long-term effects of predictors that are crucial for the assessment of the duration and the effectiveness of an intervention policy. In spite of the simplicity of the model, we find that it successfully achieves to predict the COVID-19 Daily Deaths to Daily Cases ratio.

Despite that the number of reported cases alone is not the best proxy of the actual number of cases in the community, it could be indicative of the spread of the virus, provided the consistency of the testing system in a certain region (e.g., mass testing or targeted testing, etc.). However, it is merely impossible to account for all COVID-19 cases, even in countries that adopted a mass testing strategy, like South Korea and Germany.

In order to estimate the effect of COVID-19 in terms of life loss, WHO proposes two measures: The Infection Fatality Rate (IFR)—estimating the proportion of deaths amongst all infected cases, and the Case Fatality Rate (CFR)—estimating the proportion of deaths amongst reported cases^38^. Both of these indicators can be considered accurate, if (a) the testing strategy is the same over the selected period, and (b) all active patients have either died or recovered. WHO describes CFR as “*a measure of disease severity and is often used for prognosis (predicting disease course or outcome), where comparatively high rates are indicative of relatively poor outcomes*”^38^. However, WHO proposes that, in an ongoing pandemic, CFR should be estimated as the ratio of deaths over the sum of deaths plus the recovered. In addition, the John Hopkin’s University uses the observed case-fatality ratio, defined as “*the number of deaths divided by the number of confirmed cases*”^39^.

Since Greece has not adopted the mass testing strategy and it is not possible to have knowledge of the exact number of real infections, in this paper, based on the CFR concept of using ratios, we try to find the optimal way to use the very limited data in order to perform an analysis using the resources available. We propose a multiple regression model that is independent of restrictions, and that also takes into account the active to recovered ratio, in order to provide a more complete assessment/overview of the daily spread of the regional COVID-19 epidemic.

In particular, let ${y}_{t}^{(d)}$ be the dependent variable constructed as the ratio of Daily Deaths to Daily Cases, ${x}_{i,t},$ with $i=1, 2, 3$ denoting the explanatory variables and $t=1,\dots ,T$, with $T$ being the respective number of observations. The dependent variable exhibits a series of statistical properties that pose serious challenges to standard statistical models (e.g., autoregressive fractionally integrated moving average models). For example, the autocorrelations of the square and absolute values of Daily Deaths to Daily Cases ratio display long-memory that last for long periods of time (e.g., weeks), while it is expected that its determinants will influence it after a long period of a random time.

Despite that the widely used models in the existing literature use infinite-dimension restrictions to infinite variable lags in order to be able to obtain long memory, many observations are lost because of the not time-effective build-up period for the fractional difference operator^40^. However, for cases like the one presented in our study, there is a limited number of observations as the statistical understanding is crucial to be developed over a restricted short-term period. In a sense, delays could be measured in number of casualties. What is more, such models capture the so-called unifractal scaling behavior and not the multiscaling behavior (i.e., when the data exhibits patterns that are repeated at different time scales or scaling laws). Such scaling-type regularities can provide useful information for modeling and forecasting a phenomenon.

The model developed during the implementation of this study has a simple autoregressive structure; however, highlighting the feature of considering explanatory variables over different interval sizes. The $AR(k)-{\rm H}X$ model heterogeneous is given by:

$$ \begin{aligned} {y}_{t+1}^{(d)} & =c+\sum_{i=1}^{3}{\delta }_{i}^{\left(d\right)}{x}_{i,t}+{\delta }_{1}^{\left(w\right)}\left({w}^{-1}\sum_{h=0}^{w}{x}_{1,\left(t-h\right)-w}^{(d)}\right)+{\delta }_{2}^{\left(w\right)}\left({w}^{-1}\sum_{h=0}^{w}{x}_{2,\left(t-h\right)-w}^{(d)}\right) \\ & \quad +{\delta }_{3}^{\left(w\right)}\left({w}^{-1}\sum_{h=0}^{w}{x}_{3,\left(t-h\right)-w}^{(d)}\right)+\sum_{j=1}^{k}{{\varphi }_{j}y}_{\left(t+1\right)-j}^{(d)}+{\psi D}_{t-w}^{(d)}+{\omega }_{t+1}^{(d)} \end{aligned}$$

where $c$ is the constant term, $(d)$ denotes the data frequency (i.e., daily data frequency) and $D$ is the dummy variable that is equal to one (1) for the day that an event occurs (i.e., a restriction is imposed), and zero otherwise, while $(w)$ denotes longer aggregation periods and $\left(k\right)$ is the number of lags considered in the autoregressive term. The selection of $(w)$ is data driven. In this predictability analysis, we make use of longer aggregation periods than 1 day, as we allow $\left(w\right)$ to vary over a fixed number of lags.

This data driven method allows us to find the optimal number of days passed for assessing past events that may influence the dependent variable in the future. It allows us to examine not only the effectiveness of the imposed measures via a strict statistical analysis, but also the optimal intervention framework so that such situations are predicted.

Explanatory variables viewed over different time horizons are considered, which, in turn, permit for direct comparison among quantities defined over various time horizons. This is of high significance, as it indicates how much time-in which case, days- policy makers have at their disposal in order to determine the last time-point which will allow them to act, how long such imposed measures should be in place, how the latter will be evaluated, etc. In fact, the explanatory variables are multiperiod quantities that are normalized sums of the one-period quantities (i.e., a simple average of the daily quantities).

In order to explore the relationship between the dependent and the independent variables and to avoid spurious regression results with non-stationary times series, the Augmented Dickey-Fuller (ADF) test^41,42^ and the Phillips–Perron (PP) test^43^ were used. In the case where the null hypothesis of non-stationarity (i.e., the series has a unit root) cannot be rejected, the first differences of the series are constructed. All computational analysis was performed with E-views, version 8.0.

Table 2 consists of the description of the dependent and explanatory (independent) variables used in this study.

**Table 2:** Descriptions of the dependent and independent variables for the daily deaths/daily cases model.

Variable	Description
$y$	Daily deaths to daily cases ratio
${x}_{1}$	Deaths to cases ratio^a^
${x}_{2}$	Active cases to recovered cases ratio^b^
${x}_{3}$	Google trends
$D$	Dummy for restrictive measures

^a^Refers to total deaths and total cases.

^b^Refers to total active cases and total recovered cases.

Studying the interrelations of the dependent variables measured over different time horizons, the dynamics of the different components of a system can be revealed. It is expected that interventions or infections over longer time intervals have a stronger influence on the Daily Deaths to Daily Cases ratio over shorter time intervals. Furthermore, the interpretation of the proposed model is must simpler than an autoregressive model with non-heterogenous explanatory variables taking a very high number of lags. As already mentioned, standard models employed in the literature, while possibly effective in modelling the evolution of a phenomenon that develops in an endogenous system, are not able to capture exogenous effects that have took place a long time ago (e.g., weeks or months).

### Extensions

In this section, two interesting extensions of the predictability analysis, as described above, are presented. We explore how our prediction approach behaves, if we individually consider the (a) Daily Cases and (b) Daily Deaths as the model’s dependent variables.

The $AR(k)-{\rm H}X$ model heterogeneous for Daily Cases is given by:

$$\begin{aligned} {y}_{t+1}^{(d)} &=c+{\delta }_{1}^{\left(w\right)}\left({w}^{-1}\sum_{h=0}^{w}{x}_{1,\left(t-h\right)-w}^{(d)}\right)+{\delta }_{2}^{\left(w\right)}\left({w}^{-1}\sum_{h=0}^{w}{x}_{2,\left(t-h\right)-w}^{(d)}\right)+{\delta }_{3}^{\left(w\right)}\left({w}^{-1}\sum_{h=0}^{w}{y}_{\left(t-h\right)-w}^{(d)}\right) \\ & \quad +\sum_{j=1}^{k}{{\varphi }_{j}y}_{\left(t+1\right)-j}^{(d)}+{\psi D}_{t-w}^{(d)}+{\omega }_{t+1}^{(d)} \end{aligned}$$

while, for Daily Deaths, the $AR(k)-{\rm H}X$ model heterogeneous is given by:

$$\begin{aligned} {y}_{t+1}^{\left(d\right)} &=c+\left({w}^{-1}\sum_{h=0}^{w}{x}_{1,\left(t-h\right)-w}^{\left(d\right)}\right)+{\delta }_{2}^{\left(w\right)}\left({w}^{-1}\sum_{h=0}^{w}{x}_{2,\left(t-h\right)-w}^{\left(d\right)}\right)+{\delta }_{3}^{\left(w\right)}\left({w}^{-1}\sum_{h=0}^{w}{x}_{3,\left(t-h\right)-w}^{\left(d\right)}\right) \\ & \quad +\sum_{j=1}^{k}{{\varphi }_{j}y}_{\left(t+1\right)-j}^{(d)}+{\psi D}_{t- \acute{w}}^{(d)}+{\omega }_{t+1}^{(d)}  \end{aligned}$$

In both models, $c$ is the constant term, $(d)$ denotes the data frequency (i.e., daily data frequency), and $D$ is the dummy variable that is equal to one (1) for the day that an event occurs (e.g., a restriction imposed), and zero otherwise. Furthermore, $\left(w\right)$ and $\left(\acute{w} \right)$ denote longer aggregation periods and $(k)$ is the number of lags considered in the autoregressive term. The selection of $(w)$ and $\left(\acute{w}\right)$ is data driven. In this predictability analysis, we make use of longer aggregation periods than 1 day, as we allow $\left(w\right)$ to vary over a fixed number of lags.

Tables 3 and 4 consist of the descriptions of the dependent and the independent variables for the Daily Cases and Daily Deaths models, respectively.

**Table 3:** Descriptions of the variables for the daily cases model.

Variable	Description
$y$	Daily cases
${x}_{1}$	Active cases
${x}_{2}$	Recovered cases
$D$	Dummy for restrictive measures

**Table 4:** Descriptions of variables for the daily deaths model.

Variable	Description
$y$	Daily deaths
${x}_{1}$	Active cases
${x}_{2}$	Recovered cases
${x}_{3}$	Daily cases
$D$	Dummy for restrictive measures

## Results

Greece recorded its first confirmed case on February 26th, its first death on March 12th, and imposed very strict quarantine as of March 23rd. On April 25th, the Prime Minister, Kyriakos Mitsotakis, in a public address to the Greek citizens, announced the softening of the quarantine measures as of May 4th, with some retail shops resuming operation and schools opening within the week. Special commuting forms and SMS are no longer required, and citizens are allowed to move freely but only within their Prefecture.

**Figure 3:**
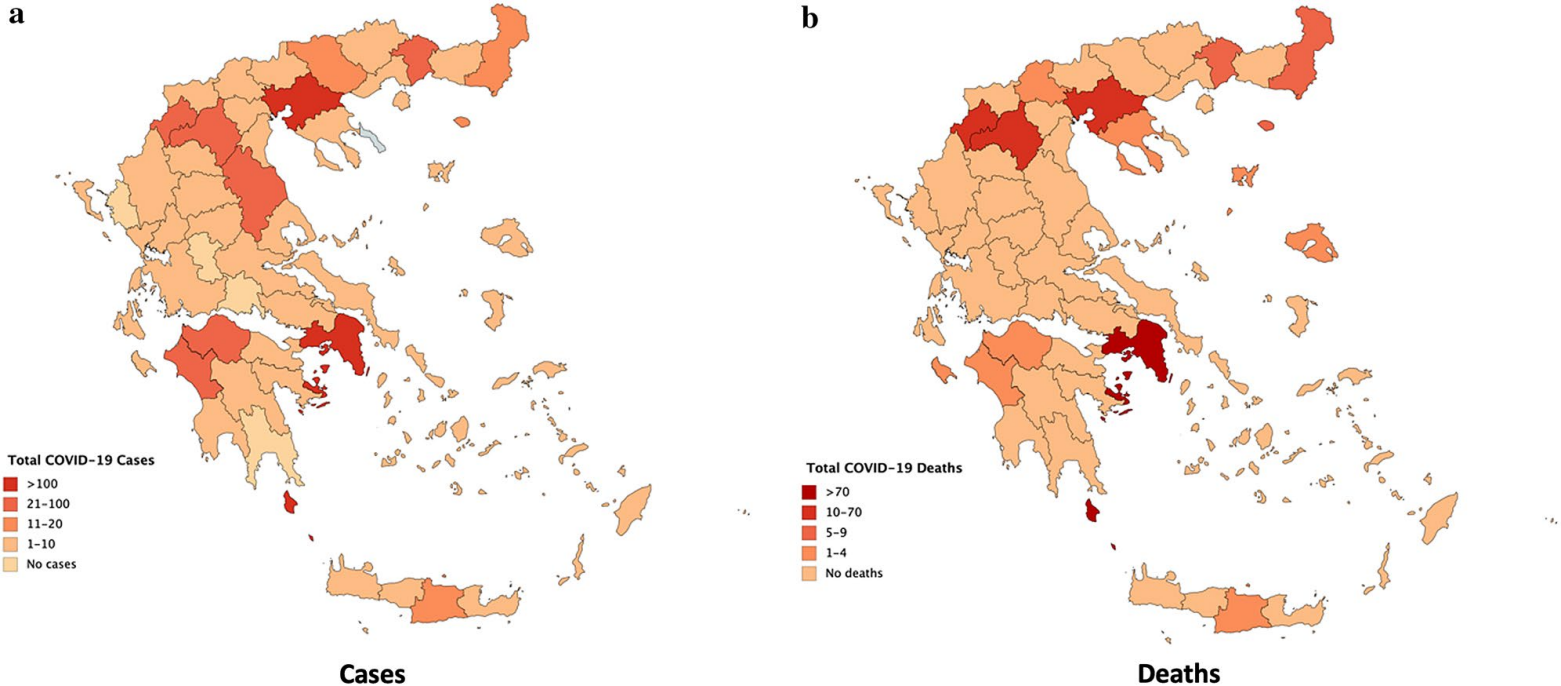
Cumulative COVID-19 (**a**) cases and (**b**) deaths in Greece as of May 3rd (Mapcharts ^23^).

The geographical distribution of the COVID-19 (a) cases and (b) deaths is depicted in Fig. 3. Up to May 3rd (4 p.m.) the country had recorded 2620 cases and 143 deaths, while 1374 COVID-19 patients had recovered. Note that some COVID-19 cases may not be included in Fig. 3a, as they do not have a geographical location (e.g., ship personnel or passengers). As is evident, Greece, with a population of 10.7 million (approx. 3.8 of which in Attica), managed to well contain the disease spread.

Figure 3b shows that most of the Greek Prefectures have recorded no deaths, while even the most affected regions (apart from Attica) only have very few casualties. This is also evident by several mainland Prefectures having recorded no COVID-19 cases, while most have recorded fewer than a total of ten confirmed COVID-19 cases.

**Figure 4:**
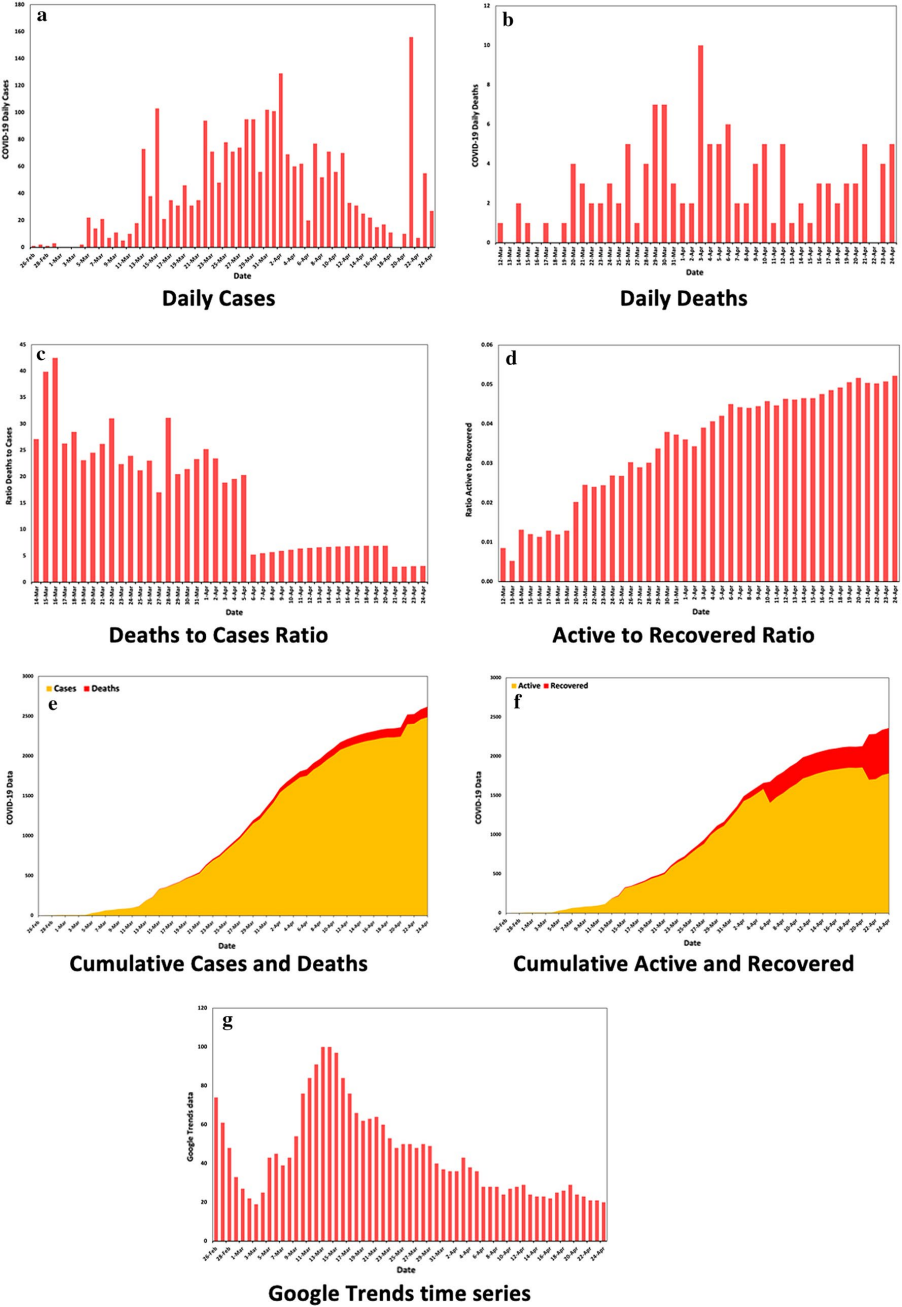
(**a**) Daily cases; (**b**) daily deaths; (**c**) deaths to cases ratio; (**d**) active to recovered ratio, (**e**) cumulative cases and deaths; (**f**) cumulative active cases and recovered; (**g**) Google Trends time series.

Figure 4 depicts the cumulative and daily COVID-19 cases, deaths, active, and recovered in Greece from February 26th to April 24th, 2020, the change in the (Deaths)/(Cases) and (Active/Recovered) ratios, and the Google Trends time series i.e., the times series used for the independent variables ${x}_{1}$, ${x}_{2}$, and ${x}_{3}$ respectively.

Note that on April 21st, a spike of 156 is observed in the daily cases (Fig. 4a); this is an outlier to the sample, as 150 of those cases were identified in a closed refugee and immigrant structure (hotel), located in a non-urban environment. As this spike was observed in a structure, it is not indicative of the spread in the community. Another spike in daily cases is observed on April 23rd, referring to a private health structure in the Attica Prefecture.

**Figure 5:**
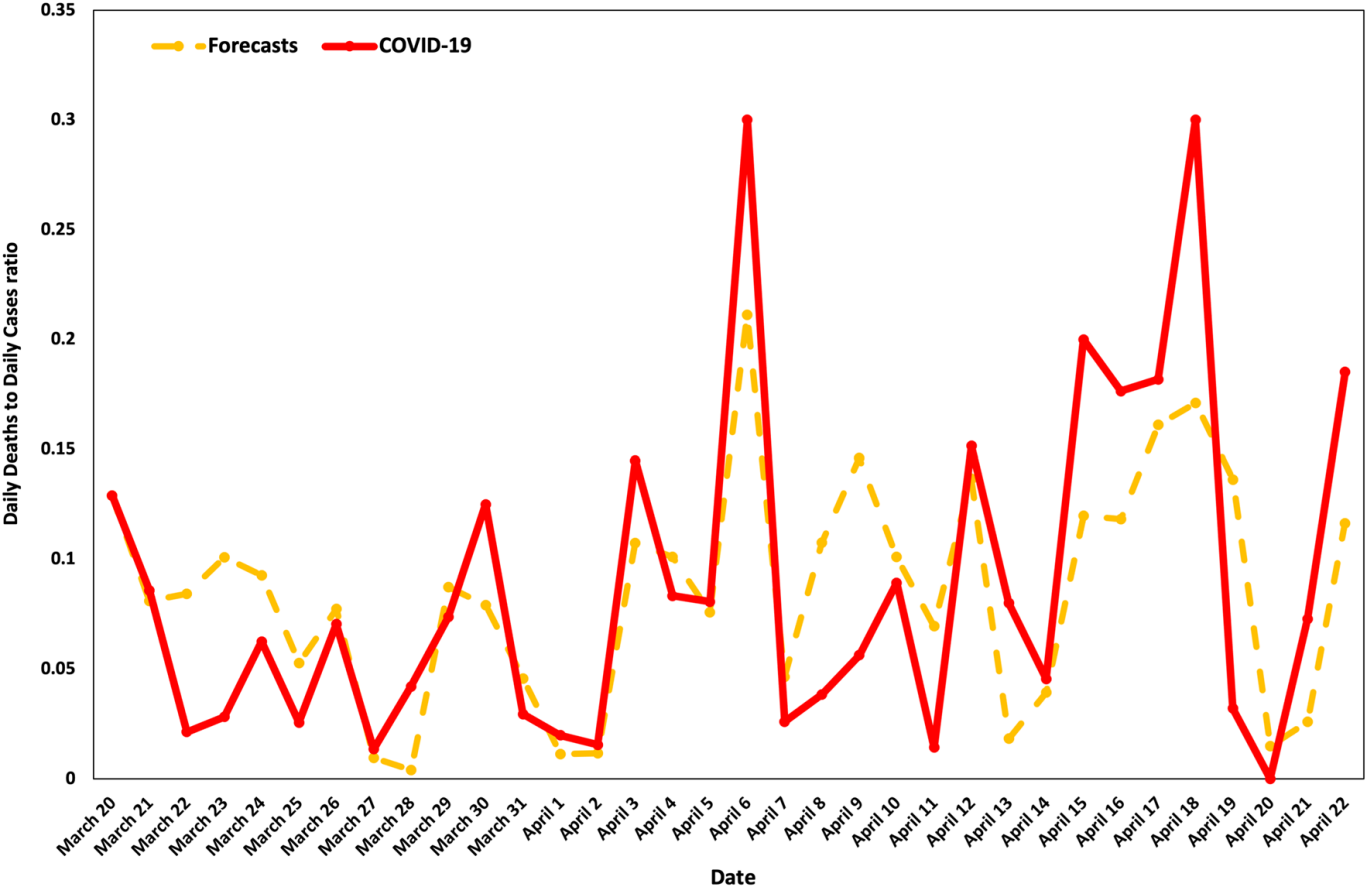
Official COVID-19 data vs. forecasts.

Figure 5 depicts the forecasts derived by the application of the $AR(2)-{\rm H}X$ model. The proposed model exhibits remarkably good forecasting performance for the Daily Deaths to Daily Cases ratio, with the latter being well predicted by the selected explanatory variables. In a sense, what our prediction model shows is the optimal time intervention framework for the flattening of the epidemic curve, in which case it is measured by the COVID-19 Daily Deaths to Daily Cases ratio.

Table 5 consists of the bootstrapped coefficient estimates and standard errors of the model with the Daily Deaths to Daily Cases ratio as the dependent variable. The application of bootstrap methods to regression models helps approximate the distribution of the coefficients and the distribution of the prediction errors.

**Table 5:** Heterogeneous autoregressive model with explanatory variables $AR(2)-{\rm H}X$ for the daily deaths to daily cases ratio.

Variable	Coefficient	Std. error	t-statistic	Prob
**Panel A. Regression estimates**				
c	0.030732***	(0.009801)	3.135473	[0.0033]
${x}_{2,t}^{(d)}$	− 0.711290***	(0.198599)	− 3.581536	[0.0009]
${7}^{-1}\sum_{h=0}^{7}{x}_{1,\left(t-h\right)-7}^{(d)}$	3.829134***	(0.536053)	7.143197	[0.0000]
${7}^{-1}\sum_{h=0}^{7}{x}_{2,\left(t-h\right)-7}^{(d)}$	2.509127***	(0.736212)	3.408159	[0.0015]
${7}^{-1}\sum_{h=0}^{7}{x}_{3,\left(t-h\right)-7}^{(d)}$	− 0.003312*	(0.001755)	− 1.887019	[0.0666]
${D}_{t-7}^{(d)}$	− 0.035046**	(0.017100)	− 2.049438	[0.0472]
${y}_{\left(t+1\right)-1}^{(d)}$	0.320404***	(0.101509)	3.156401	[0.0031]
${y}_{\left(t+1\right)-2}^{(d)}$	0.056718	(0.098270)	0.577167	[0.5671]
**Panel B. Model specification**				
Adjusted R-squared		0.597701		
Akaike information criterion		− 3.329188		
Predicted R-squared		0.429347		

***, **, and * denote statistical significance at 1%, 5%, and 10%, respectively. Bootstrapped coefficient estimates and standard errors via 2000 repetitions. The final optimal number of days (w) are selected according to the Akaike information criterion. The variance inflation factor (VIF) is used in order to deal with multicollinearity issues among the regressors. Regressors with values of VIF higher than 5 are excluded from the estimation analysis.

In this paper, we apply a bootstrap technique resampling by residuals in order to take into account the presence of influential observations (some ${x}_{t}$ very far away from the others); with the bootstrap we use an empirical distribution derived by resampling to approximate an unknown one^44^. The final optimal number of days (w) and lags (k) are selected according to the Akaike information criterion. Finally, it should be noted that, as is evident by Tables 5, 6, 7, the values of the adjusted R-squared do not have a large difference with the respective predicted R-squared, which further indicates that there is no overfitting in the models considered.

**Figure 6:**
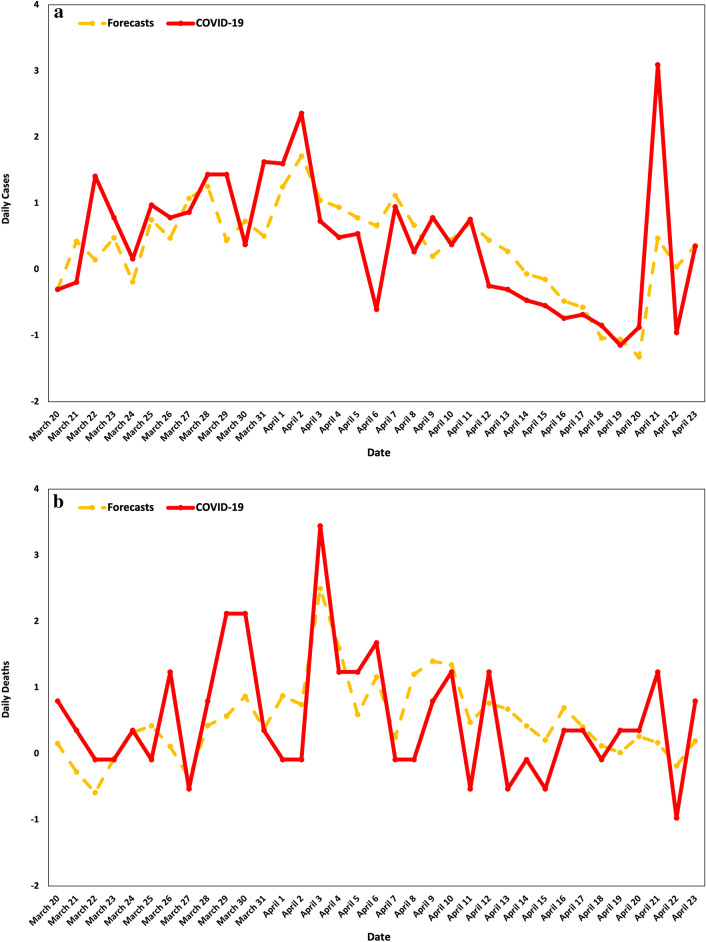
Official COVID-19 data on (**a**) daily cases vs. forecasts and (**b**) daily deaths vs. forecasts.

Furthermore, we explore how the model performs if we independently take (a) Daily Cases and (b) Daily Deaths as the dependent variable. Tables 6 and 7 present the bootstrapped coefficient estimates and standard errors of the model for Daily Cases and Daily Deaths, respectively, while Fig. 6 depicts the forecasts for the Daily Cases and Daily Deaths variables of the respective models over the examined period.

**Table 6:** Heterogeneous autoregressive model with explanatory variables $AR(1)-{\rm H}X$ for daily cases.

Variable	Coefficient	Std. error	t-statistic	Prob
**Panel A. Regression estimates**				
c	− 1.267733***	0.242387	− 5.230209	[0.0000]
${6}^{-1}\sum_{h=0}^{6}{x}_{1,\left(t-h\right)-6}^{(d)}$	0.419785***	0.128818	3.258743	[0.0021]
${6}^{-1}\sum_{h=0}^{6}{x}_{2,\left(t-h\right)-6}^{(d)}$	0.441888***	0.134237	3.291861	[0.0019]
${6}^{-1}\sum_{h=0}^{6}{y}_{\left(t-h\right)-6}^{(d)}$	− 0.368988***	0.121696	− 3.032048	[0.0040]
${D}_{t-6}^{(d)}$	− 0.557040*	0.293529	− 1.897736	[0.0640]
${y}_{\left(t+1\right)-1}^{(d)}$	− 0.212671	0.131631	− 1.615656	[0.1130]
**Panel B. Model specification**				
Adjusted R-squared		0.456847		
Akaike information criterion		2.275832		
Predicted R-squared		0.426791		

***, **, and * denote statistical significance at 1%, 5%, and 10%, respectively. Bootstrapped coefficient estimates and standard errors via 2000 repetitions. The final optimal number of days (w) are selected according to the Akaike information criterion. The variance inflation factor (VIF) is used in order to deal with multicollinearity issues among the regressors. Regressors with values of VIF higher than 5 are excluded from the estimation analysis. The variable is divided by its full-sample standard deviation, estimated based on the basic formula of the variable’s standard deviation. Therefore, the inherent variability of each variable is moved, and all variables have a standard deviation of 1.

**Table 7:** Heterogeneous autoregressive model with explanatory variables $AR(2)-{\rm H}X$ for daily deaths.

Variable	Coefficient	Std. error	t-statistic	Prob
**Panel A. Regression estimates**				
C	− 1.452676***	0.348555	− 4.167708	[0.0002]
${6}^{-1}\sum_{h=0}^{6}{x}_{1,\left(t-h\right)-6}^{(d)}$	− 0.706318***	0.151053	− 4.675970	[0.0000]
${6}^{-1}\sum_{h=0}^{6}{x}_{2,\left(t-h\right)-6}^{(d)}$	− 0.713220***	0.155345	− 4.591198	[0.0000]
${6}^{-1}\sum_{h=0}^{6}{x}_{3,\left(t-h\right)-6}^{(d)}$	0.710464***	0.147018	4.832483	[0.0000]
${D}_{t-15}^{(d)}$	− 0.678648***	0.309613	− 2.191926	[0.0348]
${y}_{\left(t+1\right)-1}^{(d)}$	− 0.273855*	0.137364	− 1.993650	[0.0536]
${y}_{\left(t+1\right)-2}^{(d)}$	− 0.392670***	0.143116	− 2.743717	[0.0093]
**Panel B. Model specification**				
Adjusted R-squared		0.424141		
Akaike information criterion		2.332387		
Predicted R-squared		0.502857		

***, **, and * denote statistical significance at 1%, 5%, and 10%, respectively. Bootstrapped coefficient estimates and standard errors via 2000 repetitions. The final optimal number of days (w) and ($\acute{w}$) are selected according to the Akaike information criterion. The variance inflation factor (VIF) is used in order to deal with multicollinearity issues among the regressors. Regressors with values of VIF higher than 5 are excluded from the estimation analysis. The variable is divided by its full-sample standard deviation, estimated based on the basic formula of the variable’s standard deviation. Therefore, the inherent variability of each variable is moved, and all variables have a standard deviation of 1.

For the Daily Cases and Daily Deaths models and based on the selection criteria, we did not include the Google Trends variable, which could be explained due to the -previously identified- decreasing trend in the online interest after some point into the pandemic^32,33^, as also evident by the negative coefficient in the Daily Deaths to Daily Cases model. Future research should explore the relationship dynamics in order to better understand and quantify the online behavioral aspects towards COVID-19.

Based on the results, for Daily Cases as the dependent variable, the optimal time intervention framework is identified at 6 days. This is supportive of the estimated incubation period as identified by the ECDC, stating that “*median incubation period from 5 to 6 days for COVID-19, with a range from two to up to 14 days*”^45^.

For Daily Deaths as the dependent variable, we need to wait 15 days in order for the NPIs to show their results. This is also supportive of previous findings, that suggest that “*the median time from illness onset to death is 18.5 days (15.0–22.0)*”^46^.

## Discussion

Battling COVID-19 is the first priority in policy planning at the moment. To that end, modeling the predictability of COVID-19 in order to minimize the spread is of great importance, at least until an effective treatment is found or a vaccine is publicly administrated and herd immunity is achieved.

In this paper, by using past COVID-19 data, an NPIs variable, and Google query data on the coronavirus, the predictability of the Daily Deaths to Daily Cases ratio in Greece is explored. On investigating the course of action in terms of NPIs of Greek officials in the COVID-19 spreading, a 7-day lag after a set of restrictive measures take place is identified. This study, apart from introducing a novel approach in the prediction of COVID-19, is a significant addition to the literature in terms of providing the direction to the appropriate course of action to minimize COVID-19 impact in terms of casualties and flattening the epidemic curve, as well as identifying that point of the regional outbreak that such NPIs should take place, in order to ensure and maximize their effectiveness.

**Figure 7:**
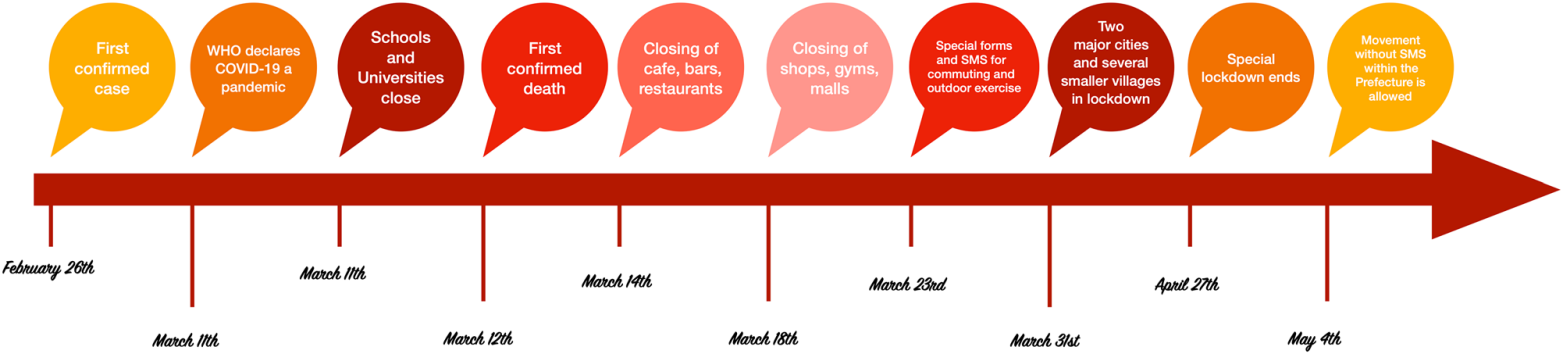
Timeline of COVID-19 in Greece.

How did the Greek government successfully handle this epidemic? What were the key milestones that they took into account in designing the operation? How were they distributed in time? Figure 7 consists of a timeline with some of the most important COVID-19 points.

The Greek officials announced the closing down of all schools and universities with the country having recorded just short of 100 cases, and only a day before the first official death was recorded on March 12th; the first COVID-19 recovery was on March 13th. The next day all cafes bars and restaurants were not allowed to seat customers, while 4 days later, all retail and gyms were prohibited from allowing customers to enter their premises; retail switched to online orders and distribution via express mail services.

Ten days after the first death, the Prime Minister in his public address, informed the Greek citizens that any outside activity is not permitted without certain special forms (or SMS), while cities and villages where regional outbreaks were identified, were put under lockdown (partial or complete). The success of the Greek case lies on the early application of any restrictive measures, which were constantly re-evaluated in order to be adjusted to any condition changes.

Greece is one of the very few examples that can be studied in order to show and validate what the real gain from applying such measures is, as it managed to do so at a very early stage. Countries that did not manage to apply NPIs at their respective appropriate time-point rather than later during the epidemic, did not minimize the spread to the point of not having grave results. Given the easy and quick spread of this virus, it should be noted that even a 48-h delay in applying NPIs could be very crucial. Slovakia is identified as another successful example during the first COVID-19 wave, adopting NPIs at a very early stage of the pandemic and while the country had not been heavily affected by COVID-19. However, Greece is an interesting case, as it is a very popular destination with many international airports that are busy throughout the year, and it also had daily connections by sea with neighboring Italy during the first weeks of the epidemic.

This is the first such approach, not only in terms of region selection, but can also assist with the assessment in policy interventions of the domestic health authorities for regionally containing the disease and minimizing the spread. The prediction model identified the optimal time-intervention framework in order to achieve the latter, with the NPIs variable being statistically significant for a 7-day lag. Therefore, countries that choose this course of action, i.e., containing the virus, should approach the subject from a multidisciplinary point of view, in order to manage (a) the softening of the measures during summer, as well as (b) the second COVID-19 wave next fall. It is understandable that an entire country cannot shut down when recording its first confirmed case, but what needs to be identified is this optimal strategy between minimizing deaths and economic and social effects at the same time.

This study has limitations. At first, a 2-month period was examined with a limited number of (daily) observations, which was, however, the case with all COVID-19 studies at that point. In addition, the NPIs variable was based on observations and recording of the events based on the authors’ experience in the examined country during the first wave of the pandemic. Moreover, an important factor that is not taken into account in this approach due to the small study period, is the number of intubated patients. This variable could provide insight in measuring the effect of the adopted NPIs, as well as in exploring the course of the pandemic during the second and third waves, where the study periods as well as the number of patients would allow for such an approach to add significant value to the modeled approaches. Finally, this prediction model takes into account four explanatory variables, while future approaches should expand this approach and aim at including more-exogenous-independent variables.

In conclusion, in this paper a 7-day lag is identified as the optimal intervention framework for the NPIs to come into effect, and this is what should be followed for successful results towards minimizing COVID-19 spreading. In particular, policy makers should take into account that NPIs that do not seem to have a positive effect on battling COVID-19 within a week, should be revised. Predicting COVID-19 is not a one-factor variable model and calls for multidisciplinary action. The present predictability analysis takes into account traditional health data (i.e., COVID-19 reported cases and deaths), specific NPIs (e.g., lockdowns), as well as infodemiology metrics (Google query data).

Our main target is to more effectively gain insight on the nature of NPIs and how the latter can assist in halting the spread. This is expected to have various consequences in the COVID-19 prediction modelling. Considering the fact that policy makers are required to make important decisions during periods with chaotic conditions, it is vital to progress with a statistical understanding of the COVID-19 time series behavior in accordance with its real determinats. In a sense, the approach should not be strictly medical in order to estimate robust COVID-19 prediction models, rather than a combination of medical and non-medical parameters from several research fields, that also take into account the citizens’ response to the measures and the way that the latter are communicated and received by the public.

## Data Availability

All data used in the analysis are open and publicly available in the cited sources.
